# Multidimensional comparative analysis of wild and cultivated *Gentiana macrophylla* based on electronic intelligent sensory technology and chemical composition

**DOI:** 10.1016/j.heliyon.2025.e42198

**Published:** 2025-01-30

**Authors:** Juanjuan Liu, Yiyang Chen, Jialing Zhang, Liangcai Wang, Ke Li, Huifang Hu, Xiaohui Ma, Ling Jin

**Affiliations:** a School of Pharmacy, Gansu University of Chinese Medicine, Lanzhou, 730000, Gansu Province, China; b Research Institute of Chinese (Tibetan) Medicinal Resources, Gansu University of Chinese Medicine, Lanzhou, 730000, Gansu Province, China; c Gansu Pharmaceutical Industry Innovation Research Institute, Gansu University of Chinese Medicine, Lanzhou, 730000, Gansu Province, China; d Northwest Collaborative Innovation Center for Traditional Chinese Medicine Co-constructed by Gansu Province *&* MOE of PR China, Lanzhou, 730000, Gansu Province, China

**Keywords:** *Gentiana macrophylla* Pall., Wild, Cultivation, Chemical constituents, Intelligent sensory technology, Multivariate statistical analysis method

## Abstract

*Gentiana macrophylla radix*, is a renowned traditional Chinese medicine, has been widely utilized in various pharmaceutical applications. The increasing demand for this material has led to the widespread cultivation of the plant. However, the differences in chemical constituents between wild and cultivated *G. macrophylla* remain unclear. In this study, an integrated approach combining a colorimeter, an electronic nose (eNose), headspace solid-phase microextraction coupled with gas chromatography-mass spectrometry (HS-SPME-GC-MS), and high-performance liquid chromatography (HPLC) was developed to investigate the chemical variations between wild and cultivated *G. macrophylla.* This method was further enhanced by multivariate statistical analysis. The fusion of intelligent sensory technologies enabled the rapid differentiation of wild and cultivated *G. macrophylla*. The chemical composition of wild and cultivated *G. macrophylla* was found to be broadly similar, with the following compounds ranked by content: gentiopicroside > loganic acid > 6-O-β-D-glucosylgentiopicroside > swertiamarin > sweroside > luteolin-6-C-glucoside > isovitexin. A total of 39 volatile components were identified, of which 17 were unique to the wild variety and 11 were unique to the cultivated variety. Nonanal, hexanal, and benzene, 1,2-dimethoxy- were identified as the primary odor-producing compounds. In summary, this study demonstrates that intelligent sensory technology, combined with chemical composition analysis, can rapidly and sensitively distinguish between wild and cultivated *G. macrophylla*. These findings are significant for the conservation of valuable genetic resources and for improving the utilization efficiency of cultivated medicinal plants.

## 1 Introduction

*Gentiana macrophylla,* a staple in traditional Chinese medicine, was first documented in *Shennong's Herbal Classic* and boasts a long history of use. The medicinal part of the plant is its dried root. Since the Tang Dynasty, *G. macrophylla Pall*. has been recognized as the primary source of *G. macrophylla.* Historical research has firmly established its origin, and it remains in use today. Wang Yuanmeng et al. suggested that this species should be prioritized in classical prescriptions involving *G. macrophylla* [1]. Characterized by its pungent, bitter and neutral taste. *G. macrophylla* is traditionally used for dispelling wind dampness, clearing damp-heat, alleviating arthralgia pain, reducing deficiency heat and treating treating a variety of conditions, including rheumatism, arthralgia, stroke-related hemiplegia, muscle spasms, aching joints, damp-heat jaundice, bone steaming hot flashes, and infantile malnutrition fever [2].

Iridoids are the primary active components of *G. macrophylla* [3]. Modern pharmacological research has shown that gentiopicroside exhibits antioxidant, anti-inflammatory, and analgesic properties. It can inhibit both acute and chronic inflammatory responses, significantly reduce serum transaminases levels in various models of acute liver and chronic liver injury, mitigate liver tissue damage (such as necrosis, swelling, and steatosis), and promote liver protein synthesis [[4], [5], [6]]. Additionally, studies have demonstrated significant differences in amino acid content between cultivated and wild *G. macrophylla*. While polysaccharide content shows no significant variation, the levels of both amino acids and polysaccharides are higher in cultivated *G. macrophylla* than in its wild counterpart [7].

Market research reveals that wild *G. macrophylla* resources are dwindling due to factors such as mixed origin, overharvesting, and limited availability. This has resulted in a small market share for wild *G. macrophylla*, with production primarily concentrated in Ningxia, Shaanxi and Shanxi provinces in China. In contrast, cultivated *G. macrophylla* holds a significant market share, particularly in Shaanxi. The decreasing availability of wild resources, rising market prices, the cultivation of high-quality varieties [8], and the standardization of large-scale planting contribute to the economic development of medicinal materials and support the growth of the Chinese herbal medicine cultivation industry [9].

Studies have shown that the chemical composition, quality and genetic diversity of Chinese herbal medicines are influenced by their growth environment and production methods [[10], [11], [12], [13]], Consequently, significant quality differences exist between wild and cultivated herbal medicines, leading to variability in clinical efficacy and substantial price fluctuations. Therefore, distinguishing between wild and cultivated herbal medicines is critical to ensuring consistent clinical efficacy and the quality of medicinal materials [14]. Current research predominantly relies on the qualitative and quantitative analysis of specific chemical index components as a quality evaluation model for traditional Chinese medicine. However, this approach does not fully capture the holistic integrity or efficacy specificity of traditional Chinese medicine [15]. Most studies are on the general plant ethnopharmacological studies. However, robust analysis like HPLC are missing in most of the studies. Therefore, this experiment adopts the multi-source information fusion evaluation model based on electronic intelligent sensory technology. It can quickly identify the wild and cultivated products of *G. macrophylla* and reflect the integrity of the quality of traditional Chinese medicine [16].

Modern electronic intelligent sensory technology offers high sensitivity, reliability, and repeatability, and repeatability in detecting quality characteristics. Correlation analyses between this technology, HS-SPME-GC-MS, and HPLS results reveal the relationship between changes in the characteristics of Chinese medicinal materials and their primary quality control indicators or overall chemical components. These findings can serve as a foundation for the quality evaluation of medicinal materials [17], particularly contributing to the quality assessment of *G. macrophylla* [18].

## 2 Materials and methods

### 2.1 Chemicals and materials

Control products: loganic acid (purity ≥98 %), 6-O-β-D-glucosylgentiopicroside (purity ≥98 %), swertiamarin (purity ≥98 %), gentiopicroside (purity ≥98 %), sweroside (purity ≥98 %), luteolin-6-C-glucoside (purity ≥98 %) and isovitexin (purity ≥98 %) were purchased from Shanghai Yuanye Biotechnology Co, LTD (China). methanol (chromatographic pure, Tianjin StarMark Science and TechnologyDevelopment Co.,Ltd, Tianjin, China), phosphoric acid (analytical pure, Shanghai Guangnuo Chemical Technology Co., Ltd, Shanghai, China), deionized water (Wahaha distilled water, Hangzhou Wahaha Group Co., Ltd, Hangzhou, China). sodium chloride (Sinopharm Group Chemical Reagent Co., Ltd. Shanghai, China), 2-Octanol (CATO, Shanghai Bohan Electronic Technology Co. , Ltd, Shanghai, China).

### 2.2 Plant materials

The sample used in this study were sourced from the authentic origins or primary producing areas of Chinese medicinal materials, ensuring their representativeness. Both wild and cultivated *G. macrophylla* samples were collected from the Qinling Mountains, China, as detailed in Table A1. A total of 10 wild samples and 10 cultivated samples were included in the study. The original plants and their roots were identified as *G. macrophylla* Pall. from the Gentian family by Professor Jin Ling of the School of Pharmacy at Gansu University of Traditional Chinese Medicine. Medium-sized herb samples were selected as test sample. The samples were dried, crushed, and sieved through a No. 4 sieve for further use.

### 2.3 Appearance character analysis

An electronic balance (XSE 205DU, Mettler-Toledo Instruments, Shanghai, China), a tape measure, and digimatic caliper (0–150 mm, Sanliang Corporation, China) were used to measure the weight, length, diameter and branches of samples. The samples consisted of naturally dried medicinal roots. To ensure accuracy and minimize errors, each measurement was performed in triplicate.

### 2.4 Colorimeter analysis

The NH310 colorimeter (Shenzhen Zhanhao Technology Co., Ltd, Shenzhen, China) was used to assess the color of the samples. The powdered sample was placed in a petridish for measurement. To minimize instrument-induced errors, a white paper standard with calibration values of L∗ = 85.779, a∗ = 6.596, and b∗ = 20.571 was used for calibration. A D65 light source was applied during the measurements. To ensure accuracy, each sample was measured three times, with three parallel measurements taken during each repetition [19]. The data L∗, a∗, and b∗ values were recorded using CQCS3 software, and the initial chromaticity values measured by the samples were used to make a difference with the standard chromaticity values, respectively, and the resulting differences were ΔL, Δa, Δb, and the difference of the three was used to find the colour difference value of ΔE= (Δa∗^2^+Δb∗^2^+ΔL∗^2^)^1/2^ [20].

### 2.5 Electronic nose analysis

#### 2.5.1 Data acquisition method analysis

In this study, The PEN3 electronic nose (eNose, Airsense company, Schwerin, Germany), containing 10 chemical sensors (Table A2), was used to preliminarily evaluate the overall aroma profile of samples [21]. The direct headspace suction method was used to set the working parameters of the electronic nose. After the stable operation of the instrument, the appropriate amount of samples was weighed and placed in a sample bottle for a fixed time. The injection needle was directly inserted into a 15 mL headspace sample bottle containing Gentiana magnolia powder at room temperature for odor data collection. The sampling time is set to 180 s, the injection flow rate is set to 600 mL min^−1^, the sensor cleaning time is set to 180 s, and the injection preparation waiting time is set to 10 s in the parameter setting.

#### 2.5.2 Determination of detection parameters

The eNose detected the fixed grinding particle size through the 4th screen, and the optimal experimental conditions were selected: incubation time was 15 min, the optimal weighing sample size was 3.0 g, and the optimal particle size was 60 mesh [22].

### 2.6 HPLC analysis

#### 2.6.1 Liquid chromatographic analysis

In this study, The High-performance liquid chromatography (HPLC) experiment was conducted using an UV detector (LC-2030C Plus, Shimadsu Malaysia Factory, Petaling Jaya, Malaysia). HPLC analysis was performed on a Zafex Supperfex RP-C18 (250 × 4.6 mm, 5 μm, Shandong Zhefen Scientific Instrument Co, Ltd, Shandong, China). The mobile phases were carbinol (A) and 0.04 % Phosphoric acid–water (B). A gradient elution was used: 35 % A at 0–5 min, 35%–40 % A at 5–10 min, 40%–45 % A at 10–15 min, 45%–60 % A at 15–20 min, and 60%–5% A at 20–25 min. The mobile phase was established at a flow rate of 1.0 mL min^−1^, the injection volume was 10 μL, and the Column temperature was 30 °C. The UV detector was used, and the detection wavelength was 242 nm. With run time was 25 min.

#### 2.6.2 Preparation of mixed reference solution

As listed in Table A3, Seven main component including loganic acid (H1), 6-O-β-D-glucosylgentiopicroside (H2), swertiamarin (H3), gentiopicroside (H4), sweroside (H5), luteolin-6-C-glucoside (H6) and isovitexin (H7). The compounds were dissolved in 70 % methanol, followed by ultrasonic treatment at a power of 100w and a frequency of 40khz using an ultrasonic cleaner (PL-FS40T, Dongguan Kangshijie Ultrasonic Technology Co., Ltd. Guangdong, China) until fully dissolved. Then, the solution was filtered through 0.22 μm membrane to prepare the mixed reference solution with concentrations of 0.140, 0.150, 0.068 1.106, 0.047, 0.011, and 0.010 mg ml^−1^, respectively.

#### 2.6.3 Preparation of sample solutions

A 1.0 g sample of No. 1–20 *G. macrophylla* medicinal powder was accurately weighed and transferred to a 50 mL volumetric flask. Next, 25 mL of 70 % methanol was added, and the initial mass was recorded. The mixture was sonicated for 40 min, allowed to cool, and reweighed. Any mass loss was compensated by adding methanol, after which the solution was thoroughly mixed and left to stand. To prepare the sample solution, 1 mL of the supernatant was transferred to a 5 mL volumetric flask, diluted with methanol, mixed well, and filtering through a 0.22 μm microporous membrane. Each sample was prepared and analyzed in triplicate.

#### 2.6.4 Methodological investigation

(1) *Linearity.* The mixed control solution was accurately measured and injected into the chromatographic system at six different injection volumes: 1 μL, 2 μL, 4 μL, 6 μL, 8 μL and 10 μL. Chromatographic conditions were as described in Section 2.6.1. The chromatographic peak areas for each compound were recorded, and a linear regression analysis was performed using the peak area (Y) as the dependent variable and the mass concentration (X) as the independent variable. A standard curve was plotted, and the linear equation was obtained.
(2) *Precision*, *Repeatability*, *Stability, and Recoveries.* The method was verified through assessments of precision, repeatability, stability and recovery. Precision was determined by calculating the relative standard deviations (RSDs) for six intra-day and inter-day measurements. Repeatability was evaluated by extracting and analyzing six replicates of the same sample, while stability was assessed by analyzing the at predefined time intervals (0, 2, 4, 8, 12, and 24 h) and calculating the RSDs for the concentrations of each analyte. Recovery was determined using spiking experiments, with the recovery rate calculated using the formula: recovery = (total detected amount − original amount)/added amount × 100 %.

### 2.7 HS-SPME-GC-MS chromatograph analysis

#### 2.7.1 GC-MS analysis

The Headspace solid-phase microextraction combined with gas chromatography-mass spectrometry (HS-SPME-GC-MS) analysis was performed on a GC apparatus coupled with MS apparatus (7890B-7000D, Agilent Technology Trade (Shanghai) Co., Ltd Shanghai, China) using HP-5MS capillary column (30 m × 0.25 mm, 0.25 μm film thickness) [23]. The analytes were separated by injecting 1 μL of a sample into an HP-5MS column. The column temperature was initially maintained at 50 °C for 2 min, then increased to 180 °C at 5 °C min^−1^ for 5 min, and then increased to 250 °C at 10 °C min^−1^ for 5 min. The inlet temperature was 250 °C, the transmission line temperature was 280 °C, and the carrier gas flow rate was 1.0 mL min^−1^. The split ratio was 50:1 [24]. The ionization mode was electron ionization (EI), and the ion source temperature was set at 230 °C. Acquisition mode is full sweep 40–600. In addition, the solvent delay was set to 2 min, and NIST 18 was used as the search library. The temperature of the four-stage rod is 150 °C.

#### 2.7.2 Pretreatment of samples

An appropriate amount of the sample was weighed and transferred into a 20 mL headspace vial. 2-octanol was added as an internal standard to achieve a final concentration of 3 mg L^−1^ on the instrument. Saturated sodium chloride was then added, and the vial was heated at 80 °C for 30 min. The headspace microextraction injection needle was inserted into the vial, with continuous heating for 30 min. The analytes were then desorbed at 250 °C for 5 min at the injection port.

### 2.8 Statistical analysis

The color and odor of *G. macrophylla* were objectively expressed by colorimeter and electronic nose technology. The results of color and odor determination of medicinal materials were combined with the characteristics of medicinal materials, the contents of chemical components determined by HPLC, and the correlation analysis was carried out. The HS-SPME-GC-MS chromatograph analysis method was used to find out the different volatile components between the two, and the quality evaluation method of *G. macrophylla* was established. The correlation between the different components, color, odor and quality of wild and cultivated *G. macrophylla* samples was analyzed.

Principal component analysis (PCA) and the orthogonal partial least squares-discriminant analysis (OPLS-DA) models were constructed using SIMCA 14.1 and Origin 2021. The data were preliminarily clustered, with the proportion of principal components serving as the primary factor. Successful clustering indicated that the model was valid. Model reliability was further verified through 200 permutation tests to evaluate potential overfitting; he absence of overfitting confirmed the model's robustness. A correlation heat map was generated using SPSS 16.0 and Origin 2021. Content comparison were performed with SPSS 16.0 and GraphPad Prism 8, while the chemical structure formulas were drawn using KingDraw. In this study, wild and cultivated species were denoted as YS and ZP, respectively.

## 3 Results and discussion

### 3.1 Appearance character results and analysis

**Fig. 1:**
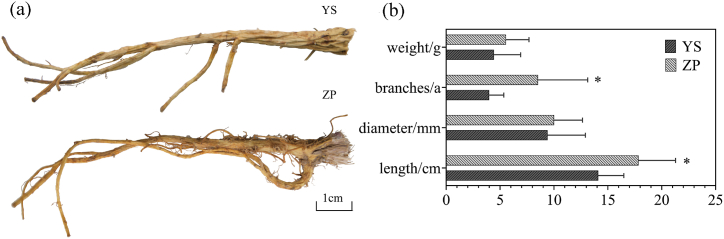
(a) Wild and cultivated *G. macrophylla* medicinal materials. (b) Comparison of main quality traits of wild and cultivated *G. macrophylla*, P ≤ 0.05.(YS: wild and ZP cultivated).

The dry weight, number of branches, diameter, and length of 20 wild and cultivated *G. macrophylla* were compared, and it was found that the values varied greatly, as shown in Fig. 1. The results indicated that the dry weight, number of branches, diameter, and length of cultivated *G. macrophylla* were greater than those of wild *G. macrophylla*. Furthermore, the length and number of branches showed significant differences between the two groups, as determined by ANOVA analysis [25].

### 3.2 Colorimeter results and analysis

**Fig. 2:**
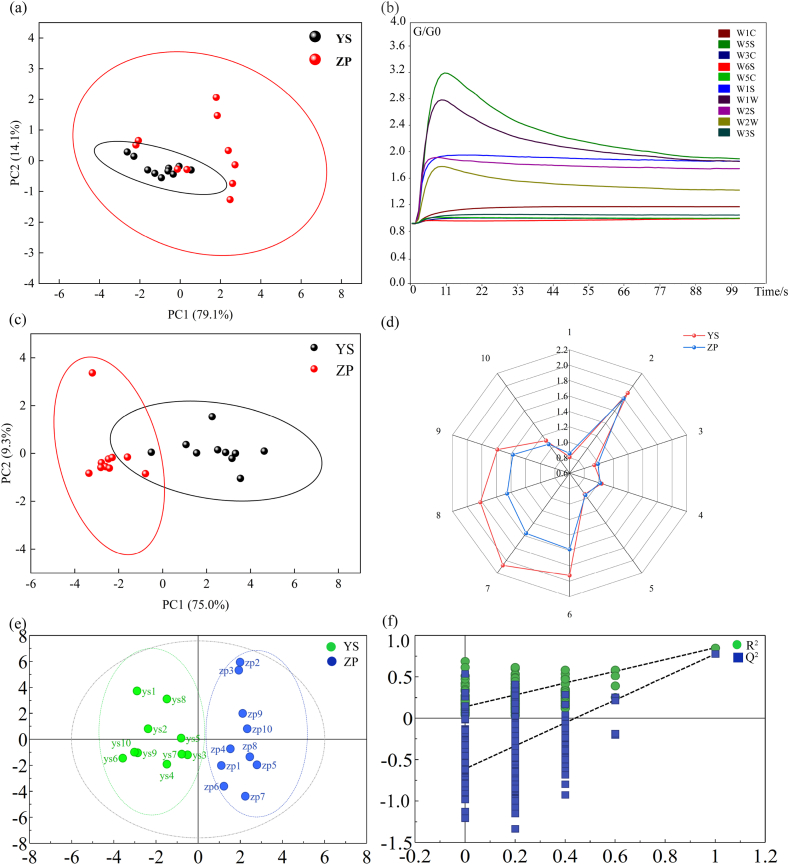
Intelligent sensory technology data analysis (a) PCA cluster map of powder Color of wild and cultivated products, (b) Response value of electronic nose for *G. macrophylla* (G/G0: electric conductivity), (c) PCA cluster map of powder odor of wild and cultivated products, (d) Radar map of wild and cultivated powder odors, (e) OPLS-DA clustering plots were analyzed for color and odor of wild and cultivated products, (f) Color and odor joint analysis of wild and cultivated products.

The colorimeter digitizes the description of the wild and cultivated color of *G. macrophylla.* It is denoted by the parameters L∗, a∗, b∗, ΔE. divides the range of wild cultivated color value, and shows that the cultivated *G. macrophylla* is yellow-brown, and the wild is light yellow. Using the measured color values L∗, a∗, b∗, ΔE as variables, the color indexes of wild and cultivated *G. macrophylla* were compared, as shown in Fig. 2 (a), the result of pca is that a single source colorimeter cannot distinguish between the two.

### 3.3 Electronic nose results and analysis

#### 3.3.1 Evaluation of assay conditions

An electronic nose (eNose) was used to collect the odor of the samples, and the responses from 10 sensors are presented in Fig. 2 (b). In this figure, each curve represents one sensor, and the points on the curves correspond to changes in the relative resistivity (G/G0, where G is the response value of the gas being measured, and G0 is the response value of air) of he sample's odor as it passes through the sensor channel over time. As shown in Fig. 2 (b), from the moment of the injection to the stabilization of the sample gas, the G/G0 ratio increased rapidly, then decreased, and ultimately stabilized. This pattern indicates that the eNose was both sensitive and stable in identifying of *G. macrophylla*, suggesting that the chemical composition of its odor is relatively stable and volatile, which can be effectively detected by the eNose.

#### 3.3.2 Odor contrastive analysis

PCA analysis of the odor data from 10 batches of wild and 10 batches of cultivated *G. macrophylla* revealed that the two groups could not be distinguished based on odor alone. However, the radar plot (Fig. 2(c) and (d)) indicated that the levels of seven components were higher in wild *G. macrophylla* compared to *G. macrophylla.* Notably, the responses from the W1S (sensitive to methane), W1W (sensitive to sulfide), W2S (sensitive to ethanol), and W2W (aromatic composition, sensitive to organic sulfides) sensors were significantly higher in wild *G. macrophylla.*

### 3.4 Combined analysis of colorimeter and electronic nose

Since a single colorimeter and eNose analysis cannot directly differentiate between wild and cultivated *G. macrophylla*., a joint sensory discrimination model was developed based on data from 20 batches of *G. macrophylla* samples, collected using both the colorimeter and eNose. and processed in SIMCA 14.1. The PCA clustering results indicated that wild and cultivated species were grouped together, which could further validated the stability of the model.

The results of the OPLS-DA identification model, shown in Fig. 2 (e), demonstrated that wild and cultivated *G. macrophylla* could be accurately classified into distinct groups. After performing 200 permutation tests, as illustrated in Fig. 2 (f), R^2^ = 0.145, Q^2^ = −0.641, and both R^2^ and Q^2^ obtained by random arrangement on the left are smaller than the original values on the right, indicating that the model is relia ble and no overfitting occurs.

### 3.5 HPLC results and analysis

#### 3.5.1 Content determination conditions analysis

**Fig. 3:**
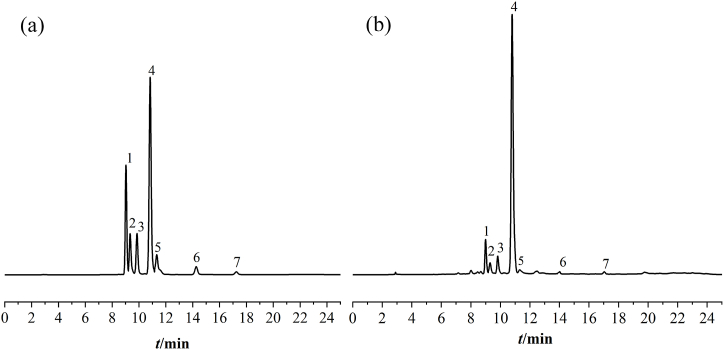
(a) HPLC chromatogramof mixed standards, (b) HPLC chromatogram of samples. (1. loganic acid; 2. 6-O-β-D-glucosylgentiopicroside; 3. swertiamarin; 4. gentiopicroside; 5. sweroside; 6.luteolin-6-C-glucoside; 7. sovitexin).

The chromatogram was recorded using HPLC, as shown in Fig. 3. The content of seven index components in 20 samples was calculated using the linear regression equation, followed by ANOVA analysis. The results indicated that the contents of H1, H2, H3, H4, H5, H6 and H7 of 10 wild and 10 cultivated *G. macrophylla* samples were determined in this study. The total content of the seven index components was higher in wild *G. macrophylla* compared to cultivated *G. macrophylla.* The component hierarchy was as follows: H4 > H1 >H2 > H3 > H5 > H6 > H7. Except for H7, the contents of the other six components were greater in the wild variety than in the cultivated one. Notably, the content of gentiopicroside was the highest, which is consistent with findings from other studies [26].

#### 3.5.2 PCA and OPLS-DA analysis

**Fig. 4:**
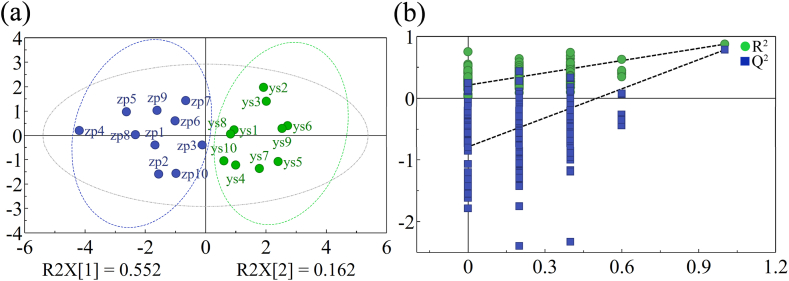
Content determination by HPLC and OPLS-DA analysis. (a) PCA analysis, (b) OPLS-DA permutation test of wild and cultivated *G. macrophylla* (n = 200).

The content of the seven components was imported into SPSS 26.0 to calculate the KMO value, which was found to be 0.732 (greater than 0.5), indicating that the data are suitable for PCA. Subsequently, the eigenvalues and variance contribution rates of the obtained variables were calculated. Factors with eigenvalues greater than 1 were selected as the conditional set for factor extraction. The analysis revealed that the information from the seven components could be reduced to two principal components, with a cumulative variance contribution rate of 71.476 %. When a threshold of 0.8 was used for factor extraction, the cumulative variance contribution rate increased to 83.082 %. the results suggest that the first three principal components effectively represent the information contained in the original variables. The principal component score plot was generated using SIMCAP 14.1 software, and the results are shown in Fig. 4 (a). The PCA diagram indicates a good clustering effect, which justifies proceeding with further OPLS-DA. The variable importance in projection (VIP) reflects the influence of each variable on the results.

Components with a VIP value greater than 1 were found to be statistically significant in the classification results. Among these components, gentiopicrin emerged as the distinguishing ingredient between wild and cultivated *G. macrophylla.* The VIP value of gentiopicrin was 2.304, highlighting its importance as a key differentiator between the two varieties. The OPLS-DA model was subjected to 200 permutation tests, and the Q^2^ intercept on the Y-axiswas found to be less than 0, confirming that the model is valid and free from overfitting. Additionally, and R^2^ value of 0.229 indicates that the model demonstrates good stability and predictive ability. The result is shown in Fig. 4 (b).

Table A4 presents the regression equations, R^2^ values, and linear ranges for the seven analytes. Excellent linear correlations were observed between the peak areas and concentrations for each analyte, with calibration curves showing good linearity (R^2^ ≥ 0.999) across the tested concentration ranges. The method developed demonstrated high sensitivity for the separation and analysis of the seven compounds.

The variations (RSD) in the precision analyses for H1, H2, H3, H4, H5, H6, and H7 ranged from 0.475 % to 3.417 %, with repeatability values between 2.569 % and 4.356 %, and stability of ranging from 0.954 % to 3.995 %. These results indicate that the precision, repeatability, and stability of the proposed method are adequate for determining the seven compounds in *G. macrophylla* samples. The average recovery rates ranged from 97.67 % to 99.57 %, with RSD values under 2 %. Therefore, the developed method demonstrated good accuracy, repeatability, and stability for the simultaneous analysis of the seven compounds.

### 3.6 HS-SPME-GC-MS results and analysis

#### 3.6.1 Common components of wild and cultivated G. macrophylla analysis

**Fig. 5:**
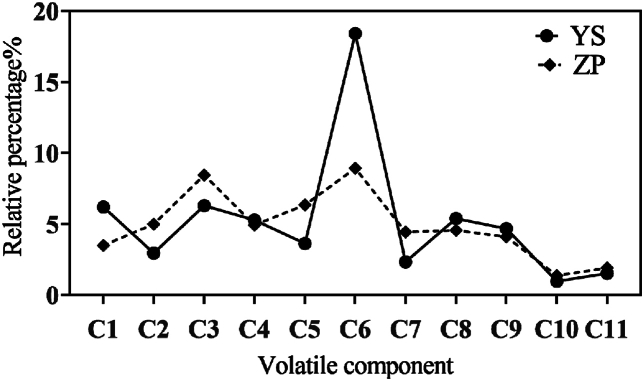
Comparison of the relative percentages of volatile oils shared by wild and cultivated *G. macrophylla*.

In the study of volatile constituents of *G. macrophylla*, 39 constituents were identified by removing duplicates and screening based on the condition that the matching factor was greater than 80 %. Among them, are 3 aromatic, 5 olefinic, 5 alcoholic, 4 aldehydes, 1 ketone, 2 carboxylic acids, 8 esters, 1 phenol, 2 ethers, 6 nitrogenous organic compounds, and 2 others. The results of the volatile oil components of wild and cultivated *G. macrophylla* are shown in Table A5, structural formula is shown in Table 1 (1), and the relative percentage contents are shown in Fig. 5, of which those that have the higher contents are esters, nitrogenous organic compounds, olefinic, aromatic, and aldehydes. Notably, cultivated *G. macrophylla* exhibited the highest content of nitrogenous constituents, while wild *G. macrophylla* had the highest content of esters [27].

**Table 1:** Volatile components of wild and cultivated *G. macrophylla*.

(1) Common volatile oil constituents of wild and cultivated *G. macrophylla*				
C1	C2	C3	C4	C5
C6	C7	C8	C9	C10
C11				
(2) Volatile oil composition and structure of wild and cultivated *G. macrophylla*				
Y1	Y2	Y3	Y4	Y5
Y6	Y7	Y8	Y9	Y10
Y11	Y12	Y13	Y14	Y15
Y16	Y17	Z1	Z2	Z3
Z4	Z5	Z6	Z7	Z8
Z9	Z10	Z11

#### 3.6.2 Unique components of wild and cultivated G. macrophylla

A total of 28 volatile constituents were identified in the 10 batches of wild *G. macrophylla*, with the three highest contents being 18.407 % C6 (erythrocentaurin), 7.650 % Y12 (phenol, 2-methoxy-3-methyl-), and 6.697 % of Y17 (methyl 9-cis,11-trans-octadecadienoate), respectively; there were 22 volatile constituents in 10 batches of cultivated herbs, and the three highest contents were 8.928 % of C6, 2-methoxy-3-(1-methyl ethyl)-Pyrazine 8.446 %, and Z1 ((E)-3-butylidene-4,5-dihydroisobenzofuran-1(3H)-one) 8.216 %.

Among them, there were 11 common components of both, namely C1 (benzene, 1,1'-[1,2-ethanediylbis(oxy)]bis-), C2 (pyrazine, 2-methoxy-3-(2-methylpropyl)-),C3 (pyrazine, 2-methoxy-3-(1-methylethyl)-), C4 (5-pentylcyclohexa-1,3-diene), C5 (benzoic acid, hydrazide), C6 (erythrocentaurin), C7 (benzene, 1,2-dimethoxy-), C8 (naphthalene), C9 (nonanal), C10 (1,3-cyclohexadiene, 1-methyl-4-(1-methylethyl)-), C11 (hexanal). C6, C1, C4, and C9 were all greater in the wild than in the cultivated, the first two being the most obvious, and the rest of the constituents were greater in the cultivated than in the wild. In addition, 17 volatile components, including Y15 (dimethyl phthalate), Y12 (nonanoic acid, methyl ester), and Y17 (benzaldehyde), are unique to wild *G. macrophylla*, and 11 volatile components, including Z6 (.gamma.-Terpinene), Z3 (.alpha.-Phellandrene), and Z5 (azulene), are unique to cultivated *G. macrophylla*, with structural formulae shown in Table 1(2).

Although both wild and cultivated *G. macrophylla* contain unique volatile components, these are primarily esters, aldehydes, alcohols, and nitrogen-containing organic compounds. Notably, 1,4-dimethoxybenzene is a unique ether component of cultivated *G. macrophylla*, which, although only weakly correlated with the index components, provides a theoretical basis for distinguishing between the two varieties.

#### 3.6.3 Electronic nose and HS-SPME-GC-MS combined PCA analysis

**Fig. 6:**
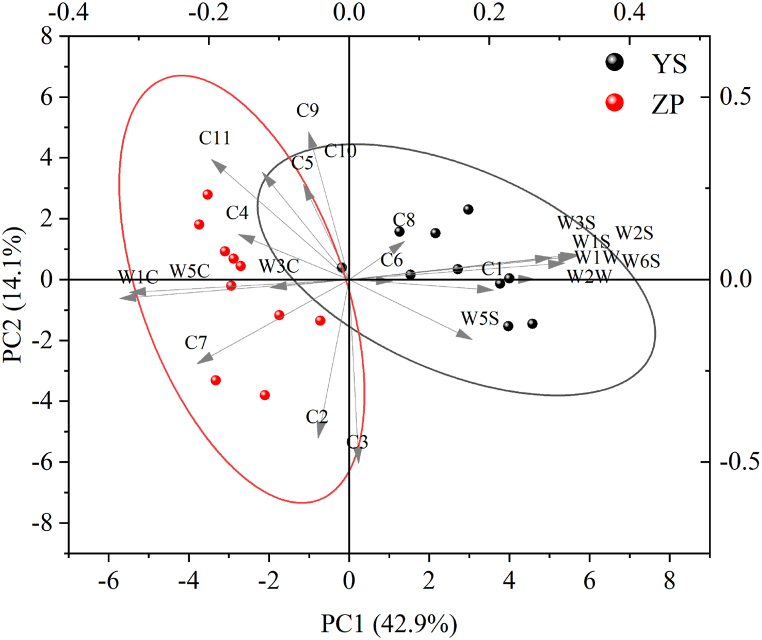
PCA correlation analysis of volatile components of wild and cultivated *G. macrophylla.*

To explore the association between the differential components and sensors of wild and cultivated *G. macrophylla*, PCA bilabial plots were performed to analyze the sexuality, and the longer arrows indicate a closer relationship with the differential components and sensors, as shown in Fig. 6. From the figure, it is evident that components such as C11, C7, C2, and C3 are closely associated with cultivated *G. macrophylla*, while the C8, C6, and C1 are linked to wild *G. macrophylla*. Additionally, components like C9, C5, C4, and C10 show a closer relationship with both varieties.

The sensors W1C, W3C and W5C are primarily aligned with cultivated *G*. *macrophylla*, with W5C showing the strongest correlation. Conversely, the remaining sensors are more closely associated with wild *G*. *macrophylla*, indicating a broader correlation with the wild variety. Notably, W1W exhibits the strongest correlation with wild *G*. *macrophylla*. Therefore, W5C and W1W can serve as key markers to distinguish between wild and cultivated *G*. *macrophylla*. This suggests that the aromatic alkane components in wild *G. macrophylla* constitute a significant proportion of the aroma, with corresponding sensor exhibiting a strong response. In contrast, cultivated *G. macrophylla* shows a strong response to the sensor sensitive to sulfide. The difference in alkane content between the two varieties are minimal, and no distinctive flavor was observed, contributing less to the aroma of the medicinal materials [28].

### 3.7 Correlation analysis based on muti-methods analysis results

#### 3.7.1 Person correlation analysis between odor measurement results and volatile oil components

**Fig. 7:**
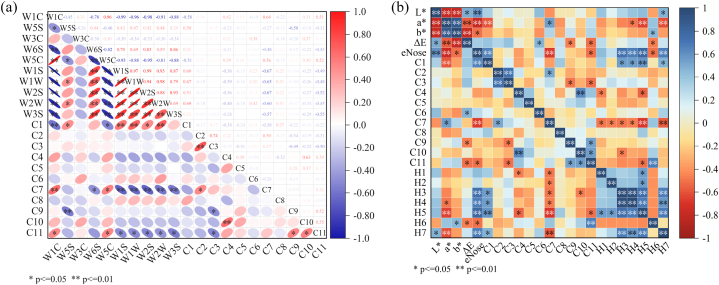
(a) Person correlation analysis of volatile components of *G. macrophylla* and electronic nose sensor (Red represents a positive correlation, blue represents a negative correlation, and white represents a non-significant correlation. The darker the color of the ovals in the figure, the stronger the correlation). (b) Fusion analysis of chemical composition and intelligent sensory information of *G. macrophylla.*

The differential compounds were analyzed through a correlation heat map with the eNose sensor data. Following radar plot analysis of eNose odor differences, all 10 sensors showed distinct variations, and thus, data from all sensors were included in the correlation analysis, as depicted in Fig. 7 (a). As shown in the figure, sensors W1C, W5C, C7 and C11 were positively correlated (P ≤ 0.05), with W1C showing a significantly positive correlation (P ≤ 0.01) with C7, Meanwhile, W1S, W1W, W2S, W2W, and W3S exhibited negatively correlated with C7 and C11, with W1C showing a significant negative correlation. Additionally, W5S showed a significant negative correlation with C9, and W6S had a negative correlation with C7. The remaining sensors were weakly correlated with the common components, as indicated by the color gradient in the figure.

#### 3.7.2 Fusion analysis of chemical composition and intelligent sensory information

The contents of chemical constituents H1, H2, H3, H4, H5, H6, and H7, along with the volatile constituents measured by gas chromatography (C1, C2, C3, C4, C5, C6, C7, C8, C9, C10, C11), were combined with organoleptic data, such as color difference values and eNose sensor information. Pearson correlation heat map analysis was conducted using Origin 2021, and the results are shown in Fig. 7 (b).

Pearson's correlation analysis revealed a significant relationship between the colour of *G. macrophylla* and the contents of H5, H6, H7, C7, and C1. Notably, a significant negative correlation was found between the contents of these components and the plant's color appearance. This suggests that the color of *G. macrophylla* can serve as a useful indicator for estimating the content of certain chemical compounds.

Moreover, the correlation analysis between the volatile components and liquid-phase results demonstrated that C4 was negatively correlated with H1 and H5. C7 exhibited a negative correlation with H1, H2, H3, H4, H5 and H7, with a significantly negative correlation to H5 and H7. There was a positive correlation with H6, but not obvious. C10 was negatively correlated with H3. C11 was negatively correlated with H5 and positively correlated with H6.

## 4 Discussion

The ecological environment significantly influences the physical characteristics of Chinese medicinal materials and the accumulation of secondary metabolites, primarily through ecological factors such as water, fertilizers, gases, and temperature [29]. For instance, the content and extraction rate of secondary metabolites in Erigeron breviscapus vary with altitudes [30]. Environmental factors also greatly affect Dendrobium officinale: for example, humidity influences the accumulation of alkaloids and flavonoids, while temperature affects the plant's height [31]. In this study, the dry weight, number of branches, diameter, and length of cultivated *G.macrophylla* were found to be higher than those of wild *G.macrophylla*, which is closely linked to the influence of these environmental factors.

Flavonoids, along with terpenoids, are key chemical constituents of *G. macrophylla*. The parent nucleus of natural flavonoids is typically substituents with groups such as-OH, -OCH3, and isopentenyl, resulting in pale yellow to yellow colors [32]. The results of this study indicated that H7 was positively correlated with the brightness value L∗, suggesting that the brighter and more yellowish the color, the higher the isovitexin content. Conversely, C1, H5, and H7 showed a significant negative correlation with the 'a' value of the red-green axis, indicating that the greener the color, the higher the content of these three constituents. A positive correlation was observed between C7 and the redness of the color, suggesting that higher ingredient content is likely associated with a redder color. H6 demonstrated a positive correlation with the yellowish-blue axis (b∗), meaning that a greater b∗value corresponds with a more yellow hue, consistent with H6 being a flavonoids. HPLC analysis revealed that the content of H7 (isovitexin) in wild *G.macrophylla* was higher than that in cultivated *G.macrophylla*, which aligns with colorimeter digitization results showing that cultivated *G.macrophylla* is yellow-brown, while wild *G.macrophylla* is light yellow.

Electronic Nose shows that the response of the sensor to the components in wild *G.macrophylla* is stronger than that in cultivated *G.macrophylla*, and that wild *G.macrophylla* is more broadly related to the sensors, indicating that there is a significant difference in odor components [33]. The sensor W5C had the strongest correlation with cultivated *G.macrophylla*. W5C was positively correlated with C7, C11 and negatively correlated with C1. W1W had the strongest correlation with wild *G.macrophylla*. W1W was negatively correlated with C7, C11 and positively correlated with C1, that is, the response values of the two were closely related to the three volatile components of C1, C7 and C11. This is closely related to the sensitivity of W5C to Aromatic components of alkane and W1W to Sensitive to sulfide. By correlation analysis based on muti-methods analysis results, it is shown that the main material basis for the odor production of *G. macrophylla* may be C7, C11 and C9.

Since a single eNose and color difference meter cannot directly and accurately distinguish wild and cultivated *G. macrophylla* [34], this study successfully visualized TCM quality by integrating two types of intelligent sensory technologies, combined with multivariate statistical and chemical composition analysis methods. The fusion of multi-source information through intelligent sensory technology and modern quality evaluation techniques for traditional Chinese medicine will foster mutual development, offering valuable insights for establishment quality markers for *G. macrophylla* [35]*.* By comparing the morphological traits and chemical compositions of wild and cultivated *G. macrophylla*, it can be concluded that cultivated *G. macrophylla* is also suitable for clinical use. Large-scale cultivation of the cultivated variety can help optimize planting methods, meet cultivation standards, reduce pressure on wild *G. macrophylla* resources, and balance market demand [36].

## 5 Conclusion and prospects

In this experimental study, it was found that the diameter, dry weight, number of branched roots, and length of cultivated *G. macrophylla* were higher than that of wild *G. macrophylla*, and the colour of cultivated *G. macrophylla* was darker, yellowish brown, and wild *G. macrophylla* was light yellow, but the content determination found that wild *G. macrophylla* was higher than that of cultivated, and the order of the content of the main components was gentiopicroside > loganic acid>6-O-β-D-glucosylgentiopicroside > swertiamarin > sweroside > luteolin-6-C-glucoside > isovitexin, and the joint analysis revealed a correlation between the colour of *G. macrophylla* and the contents of sweroside, luteolin-6-C-glucoside, isovitexin, benzene, 1,2-dimethoxy-, and benzene, 1,1'-[1,2-ethanediylbis(oxy)]bis-. Furthermore, the odor of wild *G. macrophylla* showed higher sensitivity to sensors such as W1S, W1W, W2S, and W2W compared to the cultivated variant. Gas chromatography analysis identified 39 volatile components, 17 of which were unique to wild *G. macrophylla,* and 11 were specific to the cultivated variety.

Currently, the market for wild and cultivated medicinal materials in *G. macrophylla* is mixed, with uneven quality, fluctuating prices, and a lack of rapid and accurate identification and quality evaluation methods for medicinal materials. The color and odor of medicinal materials are closely linked to their quality. In this study, intelligent sensory technology and modern quality evaluation methods for TCM were applied to integrate and analyze multi-source information. A comparison of component content between wild and cultivated *G. macrophylla* revealed slight differences, but no significant disparity. Further research is suggested from the perspective of pharmacology and pharmacodynamics to better elucidate the clinical efficacy of both types.

The use of cultivated *G. macrophylla* is beneficial for alleviating the pressure on wild resources. However, the quality of cultivated products remains inconsistent, highlighting the need for further research on quality control. It is recommended to enhance research efforts on cultivated *G. macrophylla*, expand its cultivation scale, track the growth years of plants, and prioritize the cultivation of superior species. This will provide a solid experimental foundation for the rational utilization and resource development of *G. macrophylla*.

## CRediT authorship contribution statement

**Juanjuan Liu:** Writing – original draft, Visualization, Methodology, Investigation, Formal analysis, Data curation. **Yiyang Chen:** Methodology, Investigation, Data curation. **Jialing Zhang:** Methodology, Investigation, Conceptualization. **Liangcai Wang:** Software, Investigation, Data curation. **Ke Li:** Supervision, Methodology, Investigation. **Huifang Hu:** Validation, Methodology, Data curation. **Xiaohui Ma:** Software, Resources, Investigation. **Ling Jin:** Writing – review & editing, Project administration, Methodology, Funding acquisition, Conceptualization.

## Ethics declaration

Review and/or approval by an ethics committee as well as informed consent was not required for this study because this article did not involve any direct experimentation/studies on animals.

## Data availability statement

The data presented in this study are available on request from the corresponding author.

**Table:** Abbreviations

PCA	principal component analysis
OPLS-DA	orthogonal-partial least square method-discriminant analysis
HS-SPME-GC-MS	Headspace solid-phase microextraction combined with gas chromatography-mass spectrometry
HPLC	High-performance liquid chromatography
*G. macrophylla*	*Gentiana macrophylla* Pall.

## Funding

This work was funded by China Agriculture Research System of MOF and MARA (CARS-21), Gansu Province Science and Technology Plan Project: Major Science and Technology Special (23ZDFA013-1), Gansu Provincial Science and Technology Plan Project: Science and Technology Special Touring Special (23CXNA0042), Gansu Provincial Rare Traditional Chinese Medicine Resources Evaluation and Protection and Utilization Engineering Research Center Open Fund (GSZXZY202203), Innovation Center for Traditional Chinese Medicine Co-constructed by Gansu Province & MOE of PRC, Strategic Research and Consulting Project of China Academy of Engineering (GS2021ZDA06), Improvement of traditional Chinese medicine guarantee and innovation capabilities (10.13039/501100005891State Administration of Traditional Chinese Medicine).

## Declaration of competing interest

The authors declare that they have no known competing financial interests or personal relationships that could have appeared to influence the work reported in this paper.

## Figures and Tables

**Table A1:** Sources of Gentiana macrophylla

No.	ID	Collection district	Nature
1	ZP1	Fatai Mountain, Songming Town, Hui Autonomous Prefecture, Linxia, Gansu Province	Cultivated
2	ZP2	Jiadiangou village, Fengxian County, Shaanxi province	Cultivated
3	ZP3	Nangou village, Huichuan Town, Weiyuan County, Dingxi City, Gansu Province	Cultivated
4	ZP4	Shishanping village, Liufengguan Town, Fengxian County, Shaanxi province	Cultivated
5	ZP5	Yulinpu village, Liuliufengguan Town, Shaanxi Province	Cultivated
6	ZP6	Fengjiazhuang, Liufengguan Town, Fengxian County, Shaanxi Province	Cultivated
7	ZP7	Fengxian county, Shaanxi Province, Huangniupu town Changtan Kan village	Cultivated
8	ZP8	Shadiangou Village, Fengxian County, Shaanxi Province	Cultivated
9	ZP9	Pangjiahe village, Tangzang Town, Fengxian County, Shaanxi province	Cultivated
10	ZP10	Jiadiangou village, Fengxian County, Shaanxi province	Cultivated
11	YS1	Beichenpu Town, Tongwei County, Dingxi City, Gansu Province	Wild
12	YS2	Xigou, Gupo Town, Gangu County, Tianshui City, Gansu Province	Wild
13	YS3	Songming Town, Hui Autonomous Prefecture, Linxia, Gansu Province	Wild
14	YS4	Nawu Town, Hezuo City, Gannan Tibetan Autonomous Prefecture, Gansu Province	Wild
15	YS5	Azitan Town, Zhuoni County, Gannan Tibetan Autonomous Prefecture, Gansu Province	Wild
16	YS6	Dacaotan, Mazichuan Town, Minxian County, Dingxi City, Gansu Province	Wild
17	YS7	Jivinegar Bay, Yanjing Town, Zhangxian County, Dingxi City, Gansu Province	Wild
18	YS8	Guiqing Mountain, Wuyang Town, Zhangxian County, Dingxi City, Gansu Province	Wild
19	YS9	Qingyang City, Gansu Province, Heshui County Taibai town Ma canal ditch	Wild
20	YS10	Zhaojiawan, Mapo Township, Yuzhong County, Lanzhou City, Gansu Province	Wild

**Table A2:** Composition and response performance of electronic nose sensor array

No.	sensor	property
1	W1C	Aromatic component
2	W5S	High sensitivity to nitrogen oxides
3	W3C	Ammonia, sensitive to aromatic components
4	W6S	Sensitive to hydrogen
5	W5C	Aromatic components of alkane
6	W1S	Sensitive to methane
7	W1W	Sensitive to sulfide
8	W2S	Sensitive to ethanol
9	W2W	Aromatic composition, sensitive to organic sulfides
10	W3S	Sensitive to alkanes

**Table A3:** Determination of seven components in *G. macrophylla* by HPLC

Code	Ingredient
H1	loganic acid
H2	6-O-β-D-glucopyranosyl-β-D-glucopyranoside
H3	swertiamarin
H4	gentiopicroside
H5	sweroside
H6	luteolin-6-C-glucoside
H7	isovitexin

**Table A4:** Linear equations for the 10 compounds of *G. macrophylla*

Ingredient	Regression equation	Linear range (ml/mg)	R^2^
loganic acid	y = 1.238^10^7^x-4.656^10^4^	0.0141–0.141	0.9994
6-O-β-D-glucopyranosyl-β-D-glucopyranoside	y = 7.057^10^6^x-1.799^10^3^	0.015–0.15	0.9999
swertiamarin	y = 1.450^10^7^x+6.616^10^2^	0.0068–0.068	0.9998
gentiopicroside	y = 1.100^10^7^x-5.468^10^4^	0.111–1.106	0.9999
sweroside	y = 1.130^10^7^x-3.626^10^3^	0.0048–0.048	0.9999
luteolin-6-C-glucoside	y = 1.085^10^7^x-2.068^10^3^	0.0011–0.011	0.9999
isovitexin	y = 1.167^10^7^x-2.723^10^3^	0.0022–0.011	0.9998

**Table A5:** Volatile components of wild and cultivated *G. macrophylla*

Code	Compounds	CAS	Molecular formula
C1	benzene, 1,1'-[1,2-ethanediylbis(oxy)]bis-	104-66-5	C_14_H_14_O_2_
C2	pyrazine, 2-methoxy-3-(2-methylpropyl)-	24683-00-9	C_9_H_14_N_2_O
C3	pyrazine, 2-methoxy-3-(1-methylethyl)-	25773-40-4	C_8_H_12_N_2_O
C4	5-pentylcyclohexa-1,3-diene	56318-84-4	C_11_H_18_
C5	benzoic acid, hydrazide	613-94-5	C_7_H_8_N_2_O
C6	erythrocentaurin	50276-98-7	C_10_H_8_O_3_
C7	benzene, 1,2-dimethoxy-	91-16-7	C_8_H_10_O_2_
C8	naphthalene	91-20-3	C_10_H_8_
C9	nonanal	124-19-6	C_9_H_18_O
C10	1,3-cyclohexadiene, 1-methyl-4-(1-methylethyl)-	99-86-5	C_10_H_16_
C11	hexanal	66-25-1	C_6_H_12_O
Y1	benzoic acid, methyl ester	93-58-3	C_8_H_8_O_2_
Y2	1,5-dimethyl-1-vinyl-4-hexenyl butyrate	78-36-4	C_14_H_24_O_2_
Y3	benzaldehyde, 3-methyl-	620-23-5	C_8_H_8_O
Y4	1-methyl-1H-1,2,4-triazole	6086-21-1	C_3_H_5_N_3_
Y5	2,6-dimethoxytoluene	5673-07-4	C_9_H_12_O_2_
Y6	benzoyl isothiocyanate	532-55-8	C_8_H_5_NOS
Y7	phthalan	496-14-0	C_8_H_8_O
Y8	bicyclo[4.2.0]octa-1,3,5-trien-7-ol	35447-99-5	C_8_H_8_O
Y9	bicyclo[3.2.0]hepta-2,6-diene	2422-86-8	C_7_H_8_
Y10	ethylhydroquinone	2349-70-4	C_8_H_10_O_2_
Y11	phenol, 2-methoxy-3-methyl-	18102-31-3	C_8_H_10_O_2_
Y12	nonanoic acid, methyl ester	1731-84-6	C_10_H_20_O_2_
Y13	o-methoxy-.alpha.-methylbenzyl alcohol	13513-82-1	C_9_H_12_O_2_
Y14	furfurylmethylamphetamine	13445-60-8	C_15_H_19_NO
Y15	dimethyl phthalate	131-11-3	C_10_H_10_O_4_
Y16	methyl 9-cis,11-trans-octadecadienoate	13058-52-1	C_19_H_34_O_2_
Y17	benzaldehyde	100-52-7	C_7_H_6_O
Z1	(E)-3-butylidene-4,5-dihydroisobenzofuran-1(3H)-one	81944-08-3	C_12_H_14_O_2_
Z2	hexadecanoic acid, methyl ester	112-39-0	C_17_H_34_O_2_
Z3	alpha phellandrene	99-83-2	C_10_H_16_
Z4	phenylethyl alcohol	60-12-8	C_8_H_10_O
Z5	azulene	275-51-4	C_10_H_8_
Z6	gamma-terpinene	99-85-4	C_10_H_16_
Z7	3-methoxy-2,5-dimethylpyrazine	19846-22-1	C_7_H_10_N_2_O
Z8	benzeneacetic acid methyl ester	15206-55-0	C_9_H_8_O_3_
Z9	propanoic acid, anhydride	123-62-6	C_6_H_10_O_3_
Z10	benzenemethanol, .alpha.-methyl-	98-85-1	C_8_H_10_O
Z11	1,4-dimethoxybenzene	150-78-7	C_8_H_10_O_2_
